# High-Throughput Dissolution/Permeation Screening—A 96-Well Two-Compartment Microplate Approach

**DOI:** 10.3390/pharmaceutics11050227

**Published:** 2019-05-10

**Authors:** Ann-Christin Jacobsen, Anna Krupa, Martin Brandl, Annette Bauer-Brandl

**Affiliations:** 1 Drug Transport & Delivery Group, Department of Physics, Chemistry & Pharmacy, University of Southern Denmark, 5230 Odense, Denmark; acj@sdu.dk; 2 Department of Pharmaceutical Technology and Biopharmaceutics, Faculty of Pharmacy, Jagiellonian University Collegium Medicum, 30-6088 Krakow, Poland; a.krupa@uj.edu.pl

**Keywords:** solubility, dissolution, supersaturation, solubilization, permeability, biomimetic, sink, high-throughput screening, amorphous solid dispersions

## Abstract

Early formulation screening can alleviate development of advanced oral drug formulations, such as amorphous solid dispersions (ASDs). Traditionally, dissolution is used to predict ASD performance. Here, a high-throughput approach is described that simultaneously screens drug dissolution and permeation employing a two-compartment 96-well plate. Freeze-drying from hydro-alcoholic solutions was used to prepare amorphous formulations. The screening approach was tested on amorphous and crystalline tadalafil formulations with and without Soluplus^®^. The workflow consisted of: (1) dispersion of the formulations; (2) incubation within the two-compartment plate, where a dialysis membrane separated donor (dispersed formulation) and acceptor; (3) sampling (donor and acceptor), where donor samples were centrifuged to remove non-dissolved material; and (4) quantification by UHPLC-UV. To identify optimal screening conditions, the following parameters were varied: dispersion medium (buffer/biomimetic media), acceptor medium (buffer/surfactant solutions), and incubation time (1, 3, and 6 h). Surfactants (acceptor) increased tadalafil permeation. Biomimetic medium (donor) enhanced dissolution, but not permeation, except for freeze-dried tadalafil, for which the permeated amount increased. The predictiveness was evaluated by comparing dissolution-/permeation-results with in vivo bioavailability. In general, both dissolution and permeation reflected bioavailability, whereof the latter was a better predictor. High-throughput dissolution/permeation is regarded promising for formulation screening.

## 1. Introduction

Drug candidates selected by drug discovery approaches are advanced to preclinical and clinical development by formulation efforts. For new chemical entities (NCEs), where sufficient systemic exposure cannot be achieved with standard formulations, it is inevitable to identify viable candidate-enabling formulation strategies, preferably at an early stage where very limited amounts of material is available. Recently, besides physico-chemical characterization, formulation screening in high-throughput format has gained attention to streamline the costly and challenging process of formulation design. Due to the increasing fraction of NCEs with challenging physico-chemical properties, the demand for simple, rapid, yet effective formulation screening tools has increased in recent years. In particular, poor aqueous solubility is a frequently experienced challenge that has resulted in the widespread use of candidate-enabling formulation strategies with the aim to increase the oral bioavailability of poorly soluble drug compounds. Micro- and nanoparticles, amorphous solid dispersions (ASDs) and lipid-based formulations are only few examples of the many ‘enabling formulation’ strategies available today [1]. 

Miniaturized solubility screening tools have been commonly used in early development to help select suitable solid forms and salts of the drug [2,3]. In recent years, high-throughput screening (HTS) of formulation additives, such as polymer(s), polymer/surfactant combinations and drug loading, of amorphous solid dispersions (ASDs) has also been suggested [4]. High-throughput ASD screenings can be carried out in 96-well microplate set-ups and typically investigate the miniaturized formulations’ potential to induce supersaturation (often referred to as ‘spring’) and to inhibit precipitation (often referred to as ‘parachute’). 

Traditionally, supersaturation screenings employ solvent shift or film casting. Solvent shift methods evaluate, besides the so-called kinetic solubility, precipitation inhibition in the presence of additives. Here, a drug compound is dissolved in a suitable solvent at a high concentration and is then titrated into aqueous media to induce supersaturation. The aqueous medium contains excipient(s) that may inhibit the precipitation due to the solvent shift [5,6,7]. For solvent casting, drug compound, polymer and eventually surfactant solutions are prepared using a suitable volatile organic solvent, the solutions are dispensed in various combinations and/or ratios followed by solvent evaporation to form films. The films’ solid-state and/or solubility and dissolution behavior are analyzed and serve as surrogates to predict ASD performance [4,8,9,10]. Banda et al. (2017) reported a head-to-head comparison of the two screening methods using up-scaled spray-dried ASDs for verification and summarized advantages and drawbacks of both methods [11]. 

Recently, Auch and co-workers presented a melt-based screening method. Here, a melting-step was added after preparation of the solvent-casted drug/polymer films to closer mimic the conditions of hot-melt extrusion [12]. 

Alternatives for small-scale preparation of drug/polymer films include rapid solvent evaporation using spin-coating and 2D-inkjet printing, where polarized light microscopy is used to evaluate the solid-state of the films [13,14]. Films prepared by these alternative methods can only be ranked according to their solid-state and stability upon storage. Supersaturation/precipitation kinetics in an aqueous environment are, however, difficult to predict from solid-state and stability data. 

The HTS methods described above and further high-throughput formulation screenings described in literature so far have in common that they use the ‘dissolved’ amount of drug compound as primary parameter to predict the performance of the formulation. In these pharmaceutical assessments, different ‘dissolved’ states of the drug compound are typically not distinguished. Different ‘dissolved’ states (i.e., drug molecules that are ‘molecularly’ dissolved, drug molecules that are solubilized in micelles or other supra-molecular assemblies and/or drug molecules that are complexed (e.g., cyclodextrin)) may occur due to formulation excipients and/or constituents of (biomimetic) media. These different ‘dissolved’ states may have different chemical potentials and therefore may have different impacts on formulation performance [15,16,17,18,19]. Because of this, Buckley and co-workers already in 2013 argued that a change of paradigm in biopharmaceutical evaluation of enabling formulations is needed [20]. A recent review and meta-analysis on supersaturating drug delivery systems indicated the widespread failure in predicting in vivo performance from in vitro supersaturation data [21]. It has been demonstrated that by including an absorptive compartment when investigating supersaturation/precipitation kinetics, a better formulation evaluation regarding in vivo properties can be achieved [19,22,23,24,25]. Here, removal of dissolved drug compound from the dissolution vessel decreases the degree of supersaturation, thereby decreasing the likelihood of precipitation and thus retaining the supersaturated state for a longer period.

**Figure 1:**
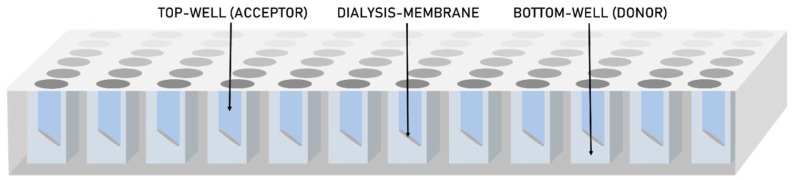
A schematic representation of the 96-well two-compartment microtiter plate.

In this work, we report a high-throughput dissolution/permeation approach for formulation screening that for the first time uses a 96-well two-compartment microplate system consisting of a conventional bottom-plate and a top-plate comprising an integrated dialysis membrane (cellulose hydrate). Figure 1 shows a schematic representation of the two-compartment microtiter plate system. This set-up was presumed to potentially solve both aforementioned challenges; differentiation between the permeation enhancing potential of molecularly dissolved and solubilized states of the drug and formulation screening in the presence of an absorptive compartment in micro-scale.

This study aimed to establish a high-throughput dissolution/permeation approach and to investigate its feasibility, practicability and predictive potential. For this purpose, formulations of the poorly soluble BCS class II drug tadalafil (TDF) were used as an example [26]. In contrast to [26], where ball milling was used, we employed freeze drying to prepare the amorphous formulations. Freeze drying resembles solvent casting yet has a minimized risk of re-crystallisation. In this study, various high-throughput dissolution/permeation experiments were conducted to investigate how different screening parameters (i.e., incubation time, dispersion media, and acceptor media) influence the practicability and predictability of the screening.

## 2. Materials and Methods 

### 2.1. Chemicals

Acetonitrile (HPLC-grade), formic acid, polysorbate 80 (PS 80), sodium chloride, sodium dihydrogen phosphate monohydrate, sodium hydrogen phosphate (anhydrous), sodium dodecyl sulfate (SDS) and tert-butanol were purchased from Sigma-Aldrich^®^ Denmark ApS (Brøndby, Denmark). Sodium hydroxide was purchased from Merck A/S (Hellerup, Denmark). Soluplus^®^, an amphiphilic graft co-polymer based on polyethylene glycol, polyvinyl caprolactam and polyvinyl acetate, was kindly donated by BASF SE (Ludwigshafen, Germany). Tadalafil (TDF; purity 99%) was purchased from abcr GmbH (Karlsruhe, Germany). D-α-Tocopheryl polyethylene glycol 1000 succinate (Vitamin E TPGS) (NF grade) was kindly donated by Gustav Parmentier GmbH (Frankfurt am Main, Germany). If not stated otherwise, chemicals were of analytical grade. Simulated intestinal fluid (SIF) instant powder containing sodium taurocholate and lecithin was purchased from biorelevant.com (London, United Kingdom). Highly purified water used for the preparation of all dispersion- and acceptor media and aqueous UHPLC solvent was freshly prepared using a Milli-Q^®^ integral water purification system (Milli-Q^®^ Advantage A10^®^; Merck Millipore, Merck A/S, Hellerup, Denmark).

### 2.2. Methods

#### 2.2.1. Preparation of Dispersion Media and Acceptor Media

In this study, three media for dispersing the tadalafil formulations were evaluated, a 50 mM phosphate buffer, simulated gastric fluid (SGF) and fasted state simulated intestinal fluid (FaSSIF). To prepare the 50 mM phosphate buffer, 1.56 g/L sodium dihydrogen phosphate monohydrate and 5.54 g/L sodium hydrogen phosphate (anhydrous) were dissolved in purified water (approximately 90% of the final volume), pH adjusted to 7.4 with 1 M NaOH and made up to the final volume. SGF was prepared according to Ph. Eur. 9.8 monograph (5.17.1. ‘Recommendations on dissolution testing’). Briefly, sodium chloride was dissolved in purified water and the pH was adjusted to 1.3 using 1 M HCl. Phosphate buffered saline (‘FaSSIF blank buffer’) pH 6.5 and FaSSIF were prepared according to ‘How to make FaSSIF/FeSSIF/FaSSGF’ (biorelevant.com 2018). FaSSIF was prepared one day before the high-throughput dissolution/permeation screening by dispersing FaSSIF instant powder in ‘FaSSIF blank buffer’. 

In this study, five acceptor media were evaluated: neat phosphate buffer and four surfactant solutions, 1% (*w*/*v*) SDS, 0.2 and 1% (*w*/*v*) Vitamin E TPGS, and 0.2 and 1% (*w*/*v*) PS 80 in phosphate buffer. Furthermore, 1% (*w*/*v*) Vitamin E TPGS solution in SGF or ‘FaSSIF blank buffer’ was prepared. All surfactant solutions had a concentration well above the respective surfactants’ critical micelle concentration. The surfactant solutions were prepared by dispersing the surfactant in the media followed by sonication at room temperature (RT) for 15 min or until a clear solution was obtained.

All dispersion- and acceptor media were stored at 4 to 8 °C for up to three months.

#### 2.2.2. Solubilization of Tadalafil in Surfactant Solutions

The solubilization of tadalafil in surfactant solutions was investigated by dispersing 4 mg tadalafil in 4 mL surfactant solution in phosphate buffer (see above). As control, 4 mg tadalafil was dispersed in neat phosphate buffer. The dispersions were shaken at 300 rpm using an orbital shaker. After 24 h, solid material was distinctively visible in all vials. Samples were withdrawn, transferred to micro-centrifuge tubes and centrifuged for 60 min at 14,000 rpm (19,500× *g*) and 25 °C using an Eppendorf Centrifuge 5804 R (Eppendorf AG, Hamburg, Germany). For quantification by UHPLC-UV, the supernatant was diluted 1:20 with a 1:1 mixture of acetonitrile and phosphate buffer.

#### 2.2.3. Preparation of Tadalafil Formulations and Their Aqueous Dispersions for High-Throughput Dissolution/Permeation Screening

Two tadalafil formulations were prepared by freeze drying, namely pure amorphous tadalafil and a 1:9 tadalafil amorphous solid dispersion (ASD) in Soluplus^®^. To prepare the formulations, tadalafil and Soluplus^®^ were dissolved in 4:1 (*w*:*w*) tert-butanol:water-mixture (tadalafil concentration 1.3 mg/mL, Soluplus^®^ concentration 3.5 mg/mL), aliquots of the solutions were transferred to 1.5 mL microcentrifuge tubes and the formulations were frozen over night at −80 °C. The Christ Gamma 2-16 LSC freeze-dryer (Martin Christ GmbH, Osterode am Harz, Germany) was pre-cooled to −60 °C and freeze drying was conducted at a pressure of 0.1 mbar and a shelf-temperature of 10 °C for 25 h. The freeze-dried formulations were placed in a desiccator over CaCO_3_ beads to reach room temperature. The high-throughput dissolution/permeation screening was conducted one day after formulation preparation. For this, 1.5 mL of the respective dispersion media was added, and the formulations were vortexed until homogenously dispersed. 

For comparison, suspensions of crystalline tadalafil and of a physical blend of tadalafil and Soluplus^®^ (further on termed ‘physical mixture’) were prepared. For this, tadalafil and a mixture of tadalafil and Soluplus^®^ were dispersed in the respective dispersion media. The tadalafil suspension and the physical mixture were stirred for 15 min at 500 rpm prior to the high-throughput dissolution/permeation experiment. Table 1 shows an overview of all prepared formulations and the concentrations of tadalafil and Soluplus^®^ in the dispersions.

#### 2.2.4. X-Ray Powder Diffraction 

The solid state of tadalafil, Soluplus^®^, freeze-dried tadalafil and the tadalafil amorphous solid dispersion was investigated by X-ray powder diffraction (XRPD) using a Rigaku^®^, MiniFlex 600 with Cu Kα radiation (l = 1.5418 Å) (Tokyo, Japan). For this, freeze-dried tadalafil and tadalafil amorphous solid dispersion were prepared in larger batches to fill the sample holder with a diameter of 2 cm. The up-scaled batches were prepared as described in Section 2.2.3. with slight modifications: a 16 mg/mL Soluplus^®^ solution was used and tadalafil and the amorphous solid dispersion were freeze dried in 100 and 30 mL vials, respectively. The XRPD analysis was carried out with a scanning rate of 1 °/min in 2θ, a step width of 0.02° over the range of 5–35°. The current was 10 mA and the voltage 30 kV. 

#### 2.2.5. High-Throughput Dissolution/Permeation Screening

**Figure 2:**
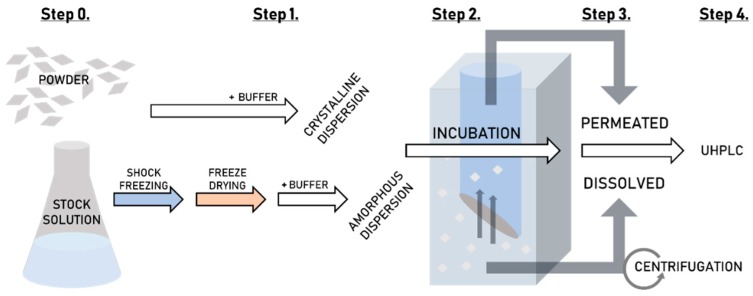
A flow-diagram of the high-throughput dissolution/permeation screening. Step 0. Formulation preparation, Step 1. Dispersion of the formulations, Step 2. Incubation of the two-compartment plate, Step 3. Sampling from the top-plate (acceptor) and bottom-plate (donor) and Step 4. Quantification of tadalafil by UHPLC.

Figure 2 shows a flow-diagram of the complete high-throughput dissolution/permeation screening procedure including preparation of the formulation, where applicable (See Section 2.2.3), the dissolution/permeation experiments (described in this section) and the quantification of tadalafil (See Section 2.2.7).

Table 2 gives an overview of all the formulations that were tested for high-throughput dissolution/permeation. In more detail, Table 2 gives an overview of the parameters under which the formulations were tested to evaluate the influence of acceptor media, incubation time and dispersion media, as well as to evaluate predictiveness of the screening by comparison to oral bioavailability data. 

The screening experiments were conducted in prototypes of a novel two-compartment microtiter plate system, which were kindly provided by InnoMe GmbH (Espelkamp, Germany). The top-plate of the microtiter plate system comprised an integrated dialysis membrane consisting of cellulose hydrate. The screening experiments were conducted according to the following general procedure: 300 µL of the freshly dispersed formulations were transferred to the bottom-plate (*n* = 3–6) and 200 µL of acceptor medium (surfactant solution or phosphate buffer) was placed in the top-wells of the plate. The top-plate was sealed with pierceable, adhesive sealing foil (x-Pierce™, Excel Scientific, Inc.) and closed with the corresponding lid. The set was incubated at room temperature under shaking (300 rpm) with an orbital shaker. During the incubation, tadalafil permeated from the bottom-wells (i.e., donor compartment), to the top-wells (i.e., acceptor compartment). After either 1, 3 or 6 h of incubation, samples were withdrawn from both, the donor and the acceptor compartments. The samples from the acceptor compartments (140 µL) were diluted 1:1 with acetonitrile for UHPLC-UV analysis. To separate the dissolved drug from the non-dissolved solid material, the samples from the donor compartments (complete well volume) were transferred to micro-centrifuge tubes and centrifuged for 60 min at 14,000 rpm (19,500× *g*) and 25 °C using an Eppendorf Centrifuge 5804 R (Eppendorf AG, Hamburg, Germany). The supernatant was diluted 1:1 with acetonitrile for UHPLC-UV analysis. Quantification of tadalafil was then conducted via UHPLC-UV as described in Section 2.2.7. The tadalafil concentration in the donor- and acceptor compartment, that is, the dissolved and permeated concentrations, were used to compare the screening parameters and to establish an in vitro in vivo correlation. The in vitro in vivo correlation was established by comparing to literature data [26]. 

#### 2.2.6. Non-Specific Adsorption of Tadalafil to Plastic Material

The loss of a drug compound due to non-specific adsorption to plastic material may represent a problem. To investigate if tadalafil adsorbs to the plastic material of the two-compartment microtiter plate a 0.9 µg/mL tadalafil solution in purified water was prepared, which is well below the aqueous solubility of tadalafil (~2 µg/mL). The tadalafil solution was transferred to the bottom-plate of the two-compartment microtiter plate system and incubated. The tadalafil concentration was determined after 3, 6 and 24 h of incubation as described in Section 2.2.7. using UHPLC-UV and compared to the start concentration. 

#### 2.2.7. Quantification of Tadalafil by UHPLC-UV

Quantification of tadalafil was conducted on a Thermo Fisher UltiMate 3000 UHPLC system that was connected to a Diode Array detector and equipped with a reversed phase Kinetex^®^ EVO C18 LC-column (100 × 2.1 mm; particle size 1.7 μm; pore size 100 Å, Phenomenex^®^). The flow rate was 0.3 mL/min and the mobile phase consisted of 57% (*v*/*v*) highly purified water containing 0.1% (*v*/*v*) formic acid as modifier and 43% (*v*/*v*) acetonitrile (isocratic elution). The column oven temperature was set to 40 °C. The total run time was 3 min, where tadalafil eluted after 1.5 min. tadalafil was detected at a wavelength of 295 nm. Two standard curves were prepared with concentration ranges of 0.1–2 µg/mL and 2–20 µg/mL, respectively. The injection volume was 5 µL.

#### 2.2.8. Statistical Analysis

Statistical analysis was performed using a one-way ANOVA test to identify statistical significance within groups of data sets and a two-tailed, unpaired Student’s t-test to compare two data sets. For both tests, *p* < 0.05 was considered as statistically significant.

## 3. Results and Discussion

### 3.1. Sample Preparation and Solid-State Analysis by X-Ray Powder Diffraction 

Krupa et al. (2016) proposed high-energy ball milling as a process to obtain amorphous tadalafil and amorphous solid dispersions of tadalafil in Soluplus^®^ by co-milling [26]. High-throughput formulation screening applications require a simple formulation preparation method, by which formulations with different excipients, excipients combinations and/or excipient ratios can be prepared, preferably in a single run. Classical (co-)milling does not meet these criteria because only one formulation can be prepared per run. We used freeze-drying from hydro-alcoholic solutions as a simple and rapid process to obtain (amorphous) tadalafil formulations for screening applications. 

**Figure 3:**
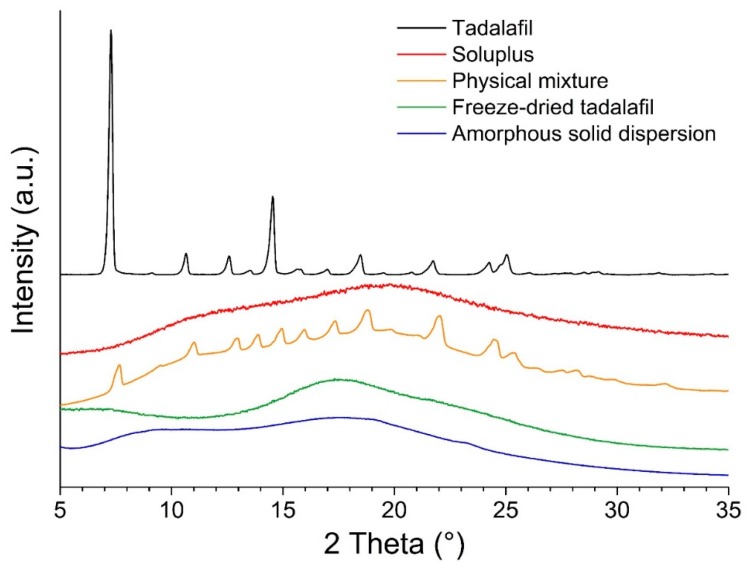
X-ray diffraction patterns of all formulations, tadalafil and Soluplus^®^.

In the diffractograms shown in Figure 3, the tadalafil-crystal specific pattern is absent in the freeze-dried samples (tadalafil alone and co-freeze dried tadalafil with Soluplus^®^). This indicates that these samples are amorphous and that freeze-drying from hydro-alcoholic solutions is a viable option to obtain amorphous formulations for screening applications.

### 3.2. Non-Specific Adsorption of Tadalafil to Plastic Material 

**Figure 4:**
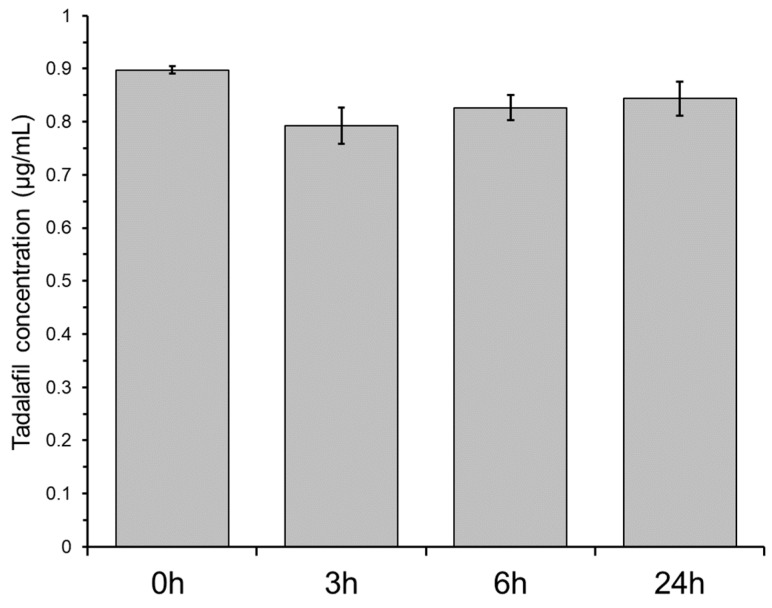
Non-specific adsorption of tadalafil to the plastic material of the two-compartment microplate system shown as the tadalafil concentration in the bottom-well after 3, 6 and 24 h incubation of a 0.9 µg/mL tadalafil solution. ‘0 h’ indicates the ‘unincubated’ (start) concentration. Data shown as mean ± SD of six replicates.

Non-specific adsorption of drug compounds to plastic material can be detrimental for biopharmaceutical assessments. This is commonly investigated by incubating a solution of the drug compound in the presence of the material. A decrease in drug compound concentration is regarded to indicate adsorption to the material. Figure 4 shows the tadalafil concentration in the bottom-wells when incubating a 0.9 µg/mL tadalafil solution. After 3 h of incubation, the tadalafil concentration is decreased by approximately 12% indicating minor adsorption to the plastic material. After 6 and 24 h of incubation, the tadalafil concentration increased slightly, likely due to solvent evaporation. Even though precautions are taken (e.g., by using sealing films), evaporation from microtiter plates is often observed due to their large surface area. The minor non-specific adsorption was regarded negligible for the further dissolution/permeation screenings. 

### 3.3. Preliminary Experiments—Incubation Time, Acceptor Media and Dispersion Media

#### 3.3.1. The Influence of Acceptor Media 

**Figure 5:**
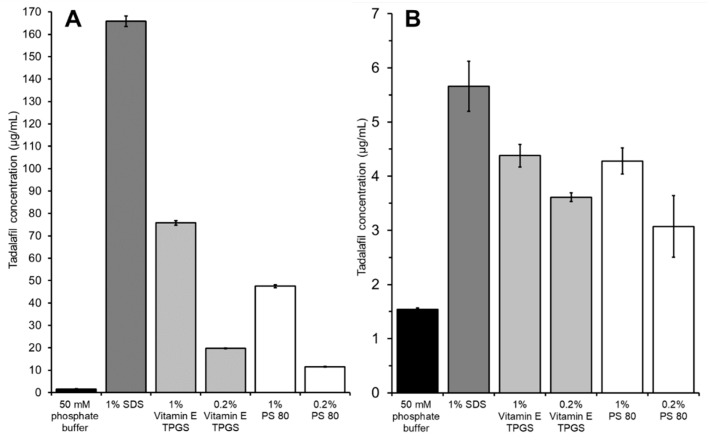
Comparison of (**A**) the apparent solubility of tadalafil in different surfactant solutions with (**B**) the tadalafil concentration in the acceptor compartment when using the different surfactant solutions as acceptor media. In all cases the donor was a suspension of crystalline tadalafil in phosphate buffer. Both (**A**) and (**B**) were determined after 24 h of incubation. Data shown as mean ± SD of triplicates.

In Figure 5 the solubilization effect of different surfactant solutions on tadalafil solubility is compared to the tadalafil permeation when using these surfactant solutions as acceptor media. The dissolution/permeation experiment was conducted using a suspension of crystalline tadalafil as donor. In both studies neat phosphate buffer served as a control, and incubation time was 24 h.

All surfactant solutions solubilized tadalafil as indicated by a significant increase in tadalafil concentration compared to neat phosphate buffer (see Figure 5A). When using these surfactant solutions as acceptor media, the permeated amount of tadalafil was increased significantly in all cases (see Figure 5B). Tadalafil solubilization and permeation enhancement seem to follow a similar pattern, where the highest solubilization leads to the fastest permeation. However, the differences in terms of solubilization are far more pronounced than the differences in permeation rate.

1% SDS yielded both the highest tadalafil solubilization and fastest permeation. When comparing the effectiveness of the other surfactant solutions, Vitamin E TPGS and PS 80, no significant difference in terms of permeation was found between the two surfactant solutions with the same concentrations (0.2% or 1%), even though a significant difference was seen in solubilization capacity. Still, a surfactant concentration of 1% compared to 0.2% was superior in both cases, which is also reflected in the solubilization capacity. 

In general, the results indicate that using surfactant solution as acceptor medium is beneficial for the high-throughput dissolution/permeation screening by enhancing the permeation of poorly soluble drug compounds. The permeation enhancing effect can be explained by at least two factors. Firstly, the surfactant solutions decrease adsorption to the plastic material. Secondly, the surfactants solubilize the drug compound in the acceptor compartment thereby maintaining/increasing the driving force for permeation. In essence, both factors result from increasing the drug compounds affinity towards the acceptor medium. 

Due to analytical challenges observed with samples containing 1% SDS (i.e., chromatograms with poor peak shape), 1% Vitamin E TPGS solution was used as acceptor medium in further high-throughput dissolution/permeation experiments. 

#### 3.3.2. The Influence of Incubation Time

**Figure 6:**
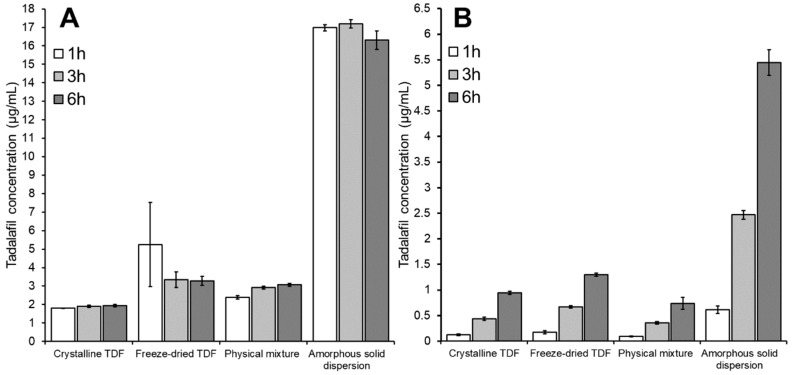
Tadalafil concentration in (**A**) the donor compartment and (**B**) the acceptor compartment after 1, 3 and 6 h of incubation when using different tadalafil formulations dispersed in 50 mM phosphate buffer as donor. The acceptor medium was 1% Vitamin E TPGS in 50 mM phosphate buffer. Data shown as mean ± SD of four replicates.

Figure 6 gives the tadalafil concentration in the donor compartment (Figure 6A) and acceptor compartment (Figure 6B) after 1, 3 and 6 h of incubation. Here, tadalafil formulations were dispersed in phosphate buffer.

As can be seen in Figure 6A, the four formulations induced different concentrations of apparently dissolved tadalafil. Focusing on the influence of incubation time in this section, this effect will be discussed in detail below (see Section 3.3.3 and Section 3.4). The different incubation times appeared to have marginal influence on the concentration of dissolved tadalafil (see Figure 6A). However, for the crystalline samples, a very slight increase in the dissolved tadalafil concentration from 1 h to 3 h was observed. This indicates that saturation solubility is almost reached after one hour in these cases. Interestingly, no change in dissolved tadalafil concentration or even a minor decrease was seen with the amorphous samples: after one hour the amorphous tadalafil (freeze-dried TDF) showed the highest dissolved tadalafil concentration with a tendency to lower values after 3 and 6 h of incubation. This can be an indication for initial supersaturation with subsequent precipitation. Due to lack of solid-state data on the precipitate, it is difficult to judge whether a precipitation in amorphous or crystalline form occurred here. For the amorphous solid dispersion high and quite constant concentrations of dissolved tadalafil were observed, which is regarded indicative for both high and long-lasting supersaturation and efficient micellar solubilization of tadalafil by Soluplus^®^ over the whole range of incubation times. 

The tadalafil concentration in the acceptor compartment (i.e., the permeated tadalafil) differed significantly between the four formulations (see Figure 6B). This effect will be discussed in more detail below (see Section 3.3.3 and Section 3.4). 

The differences between the four formulations were reflected at all three incubation times. Still, one should consider that the values after one hour were just slightly above the limit of quantitation of the analytical method (data not shown); later time points appeared thus better suited to reveal differences between the formulations in a significant manner.

#### 3.3.3. The influence of Dispersion Media 

**Figure 7:**
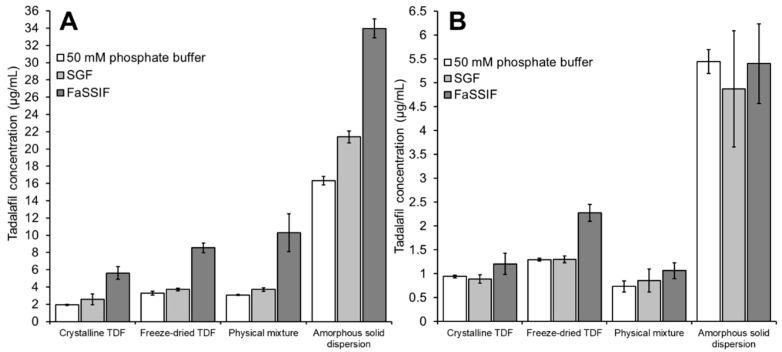
Tadalafil concentration in (**A**) the donor compartment or (**B**) the acceptor compartment after 6 h of incubation using different media for dispersion of the formulations. The acceptor medium was 1% Vitamin E TPGS in the respective dispersion medium. Data shown as mean ± SD of four replicates.

To evaluate the influence of different dissolution media on the high-throughput dissolution/permeation screening, the tadalafil formulations were dispersed in either phosphate buffer, SGF, or FaSSIF. In this screening, 1% Vitamin E TPGS dissolved in the respective medium (i.e., phosphate buffer, SGF or ‘FaSSIF blank buffer’) was used as the acceptor medium. Figure 7 shows the results from this screening test.

Figure 7A shows the tadalafil concentration in the donor compartment after six hours of incubation. For all formulations, the ranking of tadalafil concentrations in the donor compartment was FaSSIF > SGF > phosphate buffer. Here, the difference between phosphate buffer and SGF was not pronounced, whereas the tadalafil concentration in FaSSIF was approximately doubled (as compared to SGF or phosphate buffer) due to the solubilizing components, sodium taurocholate and lecithin, that are contained in this media. 

Krupa and co-workers conducted a dissolution study on similar (ball-milled) formulations using a paddle dissolution apparatus [26]. In this study, 500 mL of SGF (pH 1.2) or phosphate buffer (pH 7.4) were used as dissolution medium. After 6 h, approximately 3, 4.5, 16.6 and 24 µg/mL were reached in SGF for the dissolution of crystalline tadalafil, amorphous tadalafil, physical mixture and amorphous solid dispersion, respectively. In phosphate buffer, approximately 3, 4, 4.7 and 21 µg/mL were reached after 6 h for the same formulations. 

Compared to the large-scale dissolution method, the high-throughput dissolution/permeation method generally yielded a similar outcome in terms of dissolved tadalafil, that is, higher tadalafil concentrations in SGF as compared to phosphate buffer, higher tadalafil concentrations from amorphous tadalafil as compared to crystalline tadalafil, superior tadalafil concentrations from the amorphous solid dispersion as compared to all other formulations. 

Still, the high-throughput dissolution/permeation screening did not reflect all aspects of the large-scale dissolution study. In SGF, Soluplus^®^ positively affected the dissolution of crystalline tadalafil, that is, the tadalafil concentration of the physical mixture was approximately increased 4-fold in SGF compared to phosphate buffer, and even came close to that of the amorphous solid dispersion. The high-throughput screening results did not reflect this. Insufficient stirring of the viscous polymer solution and/or the slightly higher pH of the media (1.2 vs. 1.3) may explain this. However, in vivo oral bioavailability data from the same study did not reveal such a tremendous positive effect of Soluplus^®^ on the bioavailability of crystalline tadalafil [26]. This will be discussed in more detail below (see Section 3.4).

Furthermore, the high-throughput screening yielded lower absolute tadalafil concentrations compared to the large-scale dissolution study likely due to the temperature difference between the experiments. The high-throughput screening was conducted at room temperature (<25 °C), whereas the dissolution study was conducted at 37 °C. To increase biorelevance, the high-throughput dissolution/permeation can be conducted at 37 °C instead of 25 °C. However, increased evaporation can be a limiting factor at higher temperatures. 

Figure 7B shows the tadalafil concentration in the acceptor compartment after six hours of incubation. In contrast to the tadalafil concentration in the donor compartment (see Figure 7B), the different dispersion media did not affect the tadalafil concentration in the acceptor compartment noticeably. Only for the freeze-dried tadalafil a significant difference was observed. In this case, the presence of FaSSIF significantly increased the permeation as compared to both phosphate buffer and SGF. 

In a previous dissolution/permeation study on solid phospholipid dispersions of celecoxib, a similar effect was observed where the presence of FaSSIF promoted the permeation of freeze-dried celecoxib [27]. Upon freeze-drying, the crystalline tadalafil was amorphized as shown in the XRPD analysis (see Section 3.1). In contact with aqueous media, rapid recrystallization and precipitation of freeze-dried tadalafil may be expected due to the inherent thermodynamic instability of amorphous systems. However, data from this study on tadalafil and the previous study on celecoxib suggest that FaSSIF may retard/reduce precipitation from these very unstable amorphous systems. Precipitation inhibition is a plausible mechanistic background for the enhanced permeation of freeze-dried tadalafil because the presence of FaSSIF did not noticeably affect the permeation of crystalline tadalafil and the physical mixture of tadalafil with Soluplus^®^. The precipitation inhibiting effect of FaSSIF is, however, only evident in the absence of other precipitation inhibitors (e.g., polymers). Namely in the case of the amorphous solid dispersion, the presence of FaSSIF did not enhance tadalafil permeation compared to phosphate buffer and SGF. 

Other studies investigated the effect of simulated intestinal fluids and human intestinal fluids on supersaturation [28,29,30]. These studies relied on determining the dissolved amount via conventional separation techniques (i.e., centrifugation and/or filtration). Separation is a complex and tedious process that is prone to sampling complications (i.e., filter pore-size and centrifugation parameters can affect the amount of drug defined as dissolved). Investigating supersaturation based on the amount of drug permeated across a dialysis membrane, as presented here, can be an attractive alternative to the conventional separation approach.

Furthermore, extent and duration of supersaturation, when tested under absorptive sink conditions, may exceed that observed under static conditions [31].

### 3.4. In Vitro In Vivo Correlation: Comparing the In Vitro High-Throughput Dissolution/Permeation Screening Results to In Vivo Oral Bioavailability Results

The previous section focused on practical aspects of the high-throughput dissolution/permeation screening. This section will discuss the results of the high-throughput dissolution/permeation screening from a prediction perspective by comparing them to oral in vivo bioavailability data from [26].

The in vivo study in rats conducted by Krupa and co-workers on similar tadalafil formulations prepared by ball-milling showed that the absorption of tadalafil was significantly increased from the amorphous solid dispersion containing Soluplus^®^ [26]. In both aspects, dissolution and permeation, the high-throughput screening could reflect the superiority of the amorphous solid dispersion over the other formulations (See Figure 7). For an amorphous formulation that is not ‘stabilized’ by a precipitation inhibitor (e.g., a polymer), the amorphous tadalafil (ball-milled tadalafil) performed surprisingly well in the in vivo study. Ball-milled, amorphous tadalafil yielded the second highest AUC in the in vivo study. This was not reflected very well in the dissolution experiment conducted by Krupa and co-workers where the physical mixture of tadalafil was either superior to amorphous tadalafil (in SGF) or comparable to amorphous tadalafil (in phosphate buffer pH 7.4). The observed difference between in vitro dissolution and in vivo absorption is likely due to the more pronounced propensity of molecularly dissolved vs. micellarly solubilized drug to promote transport across an absorptive barrier. The high-throughput dissolution/permeation screening, however, was able to reflect this in certain aspects of the screening.

Table 3 gives the coefficient of determination when plotting the dissolved tadalafil concentration or the permeated tadalafil concentration (i.e., the tadalafil concentration in the donor or acceptor compartment) after 6 h of incubation against the in vivo AUC. Table 3 summarizes experiments where different dispersion media were used. Generally, for both the dissolved and the permeated tadalafil concentration, a good correlation between the in vivo and the in vitro results was achieved. 

**Figure 8:**
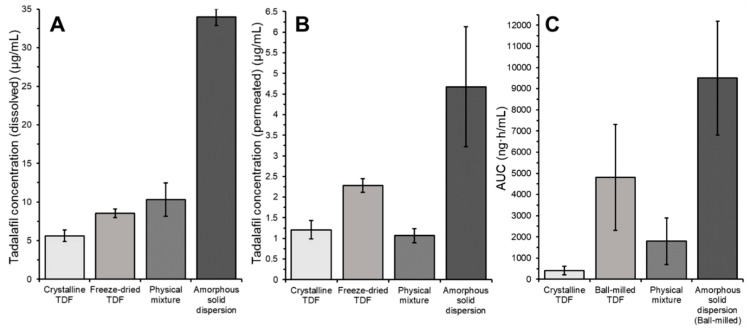
Tadalafil concentration in (**A**) the donor compartment (dissolved) or (**B**) the acceptor compartment (permeated) and (**C**) in vivo AUC after oral gavage to rats taken from [26].

Yet when SGF or FaSSIF was used as dispersion medium, the permeated tadalafil concentration correlated better with the in vivo AUC than the dissolved tadalafil concentration. For all experiments, the permeated tadalafil concentration where FaSSIF was used as dispersion medium yielded the best correlation. In these aspects of the screening, the surprisingly good performance of the amorphous tadalafil was reflected best. As discussed in Section 3.3.3, the presence of FaSSIF significantly increased tadalafil permeation from the pure amorphous formulation as compared to SGF and phosphate buffer. Experiments using SGF or phosphate buffer as dispersion medium underestimated the performance of amorphous tadalafil. Figure 8 gives an overview of the results from the high-throughput dissolution/permeation screening experiment where FaSSIF was used as dispersion medium. For comparison, the in vivo AUC’s from literature are given in Figure 8. 

The in vitro ‘absorption’ of tadalafil, that is, the amount of tadalafil reaching the acceptor compartment from the tadalafil available in the donor compartment, was relatively low as compared to the in vivo situation. For the best performing formulation, approximately 4% of available tadalafil was transported to the acceptor compartment. In this study, surfactant solutions were used to ensure sink conditions and thereby maintain a high driving-force for tadalafil permeation. 

## 4. Conclusions

In this study a novel approach was established that allows us to simultaneously investigate the dissolution (release) of a drug and its transfer across an artificial barrier over time. For this approach, a prototype 96-well titer plate with an integrated dialysis membrane separating two micro-compartments within each well was used. For evaluation of the approach, the model drug tadalafil was employed both in amorphous and crystalline state, alone and in combination with Soluplus^®^ (physical mixture and amorphous solid dispersion). In situ amorphization was accomplished by dissolution in t-butanol/water, freezing and freeze-drying. 

The concentrations of dissolved and permeated tadalafil correlated reasonably well with rat bioavailability reported in literature. A better ranking was seen for the permeated, as compared to the dissolved, amounts. Here, the good in vivo performance of pure amorphous tadalafil was best reflected. The set-up is supposed to catch the different impact that supersaturation and micellar solubilization (by the amphiphilic Soluplus^®^) have on apparent solubility and permeability. 

Among the experimental parameters tested, FaSSIF as donor medium, 1% Vitamin E TPGS as acceptor medium and sampling after six hours appeared to be superior in terms of practicability and predictiveness for the formulations investigated here. 

While the approach appears promising for early-stage high-throughput formulation performance ranking, a general conclusion regarding its predictivity, also in comparison to existing approaches, will need further evaluation, employing additional drug compounds and formulations.

## Figures and Tables

**Table 1:** Overview of tadalafil formulations/dispersions used in the high-throughput formulation screening.

Formulation	Tadalafil Concentration in Dispersion (mg/mL)	Soluplus^®^ Concentration in Dispersion (mg/mL)
Crystalline TDF	0.1	
Freeze-dried TDF	0.1	
Physical mixture	0.1	0.9
Amorphous solid dispersion	0.1	0.9

**Table 2:** Overview of all conducted high-throughput dissolution/permeation experiments to evaluate the influence of experimental parameters: (1) acceptor medium; (2) incubation time; and (3) dispersion medium.

Experimental Parameter to be Evaluated	Formulations	Dispersion Media	Acceptor Media	Incubation Time	Number of Experiments
Acceptor medium	Crystalline tadalafil (TDF)	50 mM phosphate buffer	50 mM phosphate buffer	24 h	6
			1% sodium dodecyl sulfate (SDS) in phosphate buffer		
			0.2% Vitamin E TPGS in phosphate buffer		
			1% Vitamin E TPGS in phosphate buffer		
			0.2% PS 80 in phosphate buffer		
			1% PS 80 in phosphate buffer		
Incubation time	Crystalline TDF	50 mM phosphate buffer	1% Vitamin E TPGS in phosphate buffer	1 h	12
	Freeze-dried TDF			3 h	
	Physical mixture			6 h	
	Amorphous solid dispersion				
Dispersion medium	Crystalline TDF	50 mM phosphate buffer	1% Vitamin E TPGS in phosphate buffer	6 h	12
	Freeze-dried TDF	Simulated gastric fluid (SGF)	1% Vitamin E TPGS in SGF		
	Physical mixture	FaSSIF	1% Vitamin E TPGS in FaSSIF		
	Amorphous solid dispersion				

**Table 3:** Coefficient of determination when plotting the dissolved tadalafil concentration or the permeated tadalafil concentration (i.e., concentration in donor or acceptor compartment) for experiments using different dispersion media after 6 h of incubation against in vivo AUC from [26].

Dispersion Medium	*R*^2^ (Dissolved Concentration vs. AUC)	*R*^2^ (Permeated Concentration vs. AUC)
50 mM Phosphate buffer	0.846	0.850
SGF	0.826	0.864
FaSSIF	0.823	0.940
